# Polycystic Ovary Syndrome in University Students: Occurrence and Associated Factors

**Published:** 2014-11-01

**Authors:** Amita Attlee, Asma Nusralla, Rashida Eqbal, Hanaa Said, Mona Hashim, Reyad Shaker Obaid

**Affiliations:** Department of Clinical Nutrition and Dietetics, College of Health Sciences, University of Sharjah, Sharjah, United Arab Emirates

**Keywords:** PCOS, Body Composition, Menstrual, Ultrasound

## Abstract

**Background:**

The aim of this study was to assess the occurrence of polycystic ovary syndrome (PCOS) and its association with body composition among students in University of Sharjah (UOS).

**Materials and Methods:**

This cross-sectional study included a total sample size of 50 female students registering in undergraduate programs at the University of Sharjah using convenience sampling technique. A pretested interview schedule was administered to elicit information pertaining to personal background and medical history related to PCOS. A diag- nostic ultrasound scan was performed for determining PCOS along with a body composition analysis using bioelectrical impedance analysis (BIA) technology.

**Results:**

Twenty percent (10 out of 50 participants) were diagnosed with PCOS, of whom only 4 individuals were previously diagnosed with PCOS and aware of their conditions, while the reports showed 16% with oligomenorrhea, 4% with polymenorrhea, and none with amen- orrhea. A positive family history was indicated as reported by 22% of the total participants. Significant difference between the body weights of participants having PCOS (66.7 kg) and those without it (58.8 kg) were noted (p=0.043, t=2.084). On the other hand, the body compo- sition related variables including waist-hip ratio (WHR), fat-free mass (FFM), percent body fat (PBF) and visceral fat area (VFA) were relatively higher in participants having PCOS than those without it. However, there was no statistical significance of differences. Comparatively, the participants with PCOS had lower bone mineral density (BMD) than those without it, whereas the difference was statistically non-significant.

**Conclusion:**

The occurrence of PCOS in the present study is consistent with the global preva- lence. Comparatively, the body composition of PCOS females is different from the normal fe- males. Further studies are required in the Middle East region on larger sample sizes and broader aspects of health including lifestyle and dietary components to understand these differences.

## Introduction

Polycystic ovary syndrome (PCOS) is a disorder in the function of an endocrine gland that affects the ovaries (1), involves hyperandrogenism and diminishes reproductive function (2). The disease affects around 1 in 10 women, making it the most common endocrine disorder amongst women of reproductive age (3). Some of the clinical manifestations of this disorder are irregular menstruation, infertility, weight gain, hirsutism, and acne. Also, the biochemical diagnostic features of PCOS include anovulation, insulin resistance, and hyperandrogenaemia (4). Thus, PCOS would likely increase a woman’s chance of having diabetes mellitus, hypertension and inflammation. It has been demonstrated that women with PCOS may have higher risks of cardiovascular, sleep apnea and infertility (5-8). Diagnosis of PCOS is usually based on typical signs and symptoms, physical appearance, biochemical evidence of hyperandrogenism and ovarian dysfunction (9). An ultrasound examination of the uterus/ovaries is the most reliable technique used due to morphological diagnosis of polycystic ovaries (10).

Considering the magnitude and consequences of PCOS compounded by the social apprehensions related to the nature of problem, it is important to assess its occurrence in the young adults. University female students constitute a homogenous group of population whose outreach is feasible and they are the future mothers of the society. Ironically, university students may appear healthy and not realize that they have PCOS until problems in conceiving are encountered during marriage. Lack of information about association of PCOS with other health parameters and unawareness of its diagnostic criteria may have a major impact on the presence of this disease among university females. Therefore, the objectives of the present study were to assess the occurrence of PCOS and to study its association with body composition among female students at University of Sharjah, United Arab Emirates.

## Materials and Methods

A cross-sectional study was conducted at University of Sharjah in United Arab Emirates between January 2012 and June 2012. All female students registering in undergraduate programs at the University of Sharjah were included in the present study. However, those students who were pregnant at the time of the survey period were excluded. Accordingly, fifty female students were selected using the convenience sampling technique (11). Objectives of the study and assessment needed were explained, and an informed consent was then obtained from all the participants. Furthermore, the study was approved by the Research Committee of Department of Clinical Nutrition and Dietetics, College of Health Sciences, University of Sharjah.

A pretested interview schedule was administered to collect information from the subjects. Herein, the students provided demographic information that included personal information (age, college and marital status); medical history related to PCOS; status of menstrual cycle like normal (bleeding at intervals between 22 to 40 days intervals), oligomenorrhea (bleeding at intervals of greater than 40 days) and polymenorrhea (bleeding at intervals of less than 22 days); use of hormonal pills; family history of PCOS; perception of body weight; and attempt to lose weight. Body composition data were also collected systematically at the initial clinic visit.

The required measurements were taken as follows: i. body composition of the participants was determined using the bioelectrical impedance technology (Biospace Co. Ltd., Seoul, Korea); ii. body mass index (BMI) was calculated in kg/m2 and defined according to World Health Organization (WHO) (12); iii. waist-hip ratio (WHR) was determined by the waist circumference divided by the hip circumference; iv. fat free mass (FFM) was determined by fat free mass including weight of skin, bones, ligaments, tendons, organs and water content; v. percent body fat (PBF), defined according to Li et al. (13) was calculated by the amount of fat in the body composition; vi. visceral fat area (VFA; the area in cm2 of organ fat or intra-abdominal fat) is located inside the peritoneal cavity, packed in between internal organs and torso, as opposed to subcutaneous fat found underneath the skin and intramuscular fat‚ which is interspersed in skeletal muscle (14); vii. bone mineral density (BMD; gram per square centimeter) is the bone mass after developmental period is completed (15). BMD was also measured using the Body Composition Analyzer.

Polycystic ovary was defined as the presence of at least 1 ovary at >10 cm^3^ in volume and/or at least 1 ovary with ≥12 follicles that measured 2-9 mm in diameter. Ovarian assessments were made using an ultrasound instrument (Siemens, Erlangen, Germany). The procedure of the instrument manufacturer was followed.

### Statistical analysis

Data obtained were statistically analyzed using Statistical Package for the Social Science (SPSS: SPSS Inc., Chicago, IL, USA) software version 17. Descriptive data were reported as means ± SD. Demographic and medical history variables were expressed in frequencies and percentages. Significance of difference in the variables between participants with or without PCOS was determined using student’s t test. A p value of less than 0.05 was considered to be statistically significant.

## Results

Demographic characteristics of the participants are given in table 1. Among participants, 72% were from the medical and health sciences colleges and 28% were from other different colleges. The age of the participants ranged from 17 to 23 years with the mean age of 19.4 years. Only one out of 50 participants was married. Her gynecology history revealed that she had para 1- one live child.

**Table 1:** Demographic characteristics of participants (n=50)


Variable	Participants %

**Colleges**	
Medical and health colleges	72 (36)
Other colleges	28 (14)
**Age (Y)**	
(17-19)	58 (29)
(20-23)	42 (21)
**Marital status**	
Unmarried	98 (49)
Married	2 (1)


The distribution of participants according to medical history related to PCOS is presented in table 2.

Medical history revealed that the status of menstrual cycle was normal in majority (80%) of the participants, while the finding showed that 16% had oligomenorrhea, 4% had polymenorrhea, and none had amenorrhea. Out of these, 8% had been dealing with PCOS for more than two years, 6% for over a year, and another 4% for less than 6 months. Glucose intolerance was reported in 2%, while 14% described other associated problem, specified as anemia. Twelve percent of participants took hormone pills for regularizing their menstrual cycles. While 8% of participants were on the treatment for less than 6 months, 4% were on treatment between 6 months to a year.

Ironically, only 8% of participants were previously diagnosed with PCOS, 76% had not been diagnosed earlier, and 16 % were unaware of any previous diagnosis of PCOS. About 22% showed to have the positive family history, 76% had no family history, and 2% were unaware of their family history regarding occurrence of PCOS. Amongst the individuals with positive family history, 8% reported in their mothers and sisters, 4% in cousins and 6% in their aunts. Thirty percent of participants reported to have difficulties in maintaining normal weight. When enquired about their perception of body weight, two-third of them confessed that they perceived their body weight as "normal", 14% as "underweight", 18% as "overweight" and 4% as "obese".

During the last one year, weight loss was attempted by almost half of the participants (n=24). Out of these, 16 % sought out professional support for losing weight during this period. The ultrasound scan results confirmed the diagnosis of PCOS in 10 out of 50 participants (20%).

The means and standard deviations of body composition variables of the participants are represented in table 3. In addition, significance of difference between the group with PCOS and that without PCOS is presented for each variable.

The weight of the participants ranged from 39 kg to 98 kg, with a mean weight of 60 ± 11 kg. Participants with PCOS (66.7 kg) were found to be significantly heavier than those without it (58.8 kg) (p=0.043, t=2.08).

Mean BMI of the participants was 22.9 ± 3.5 ranging from 16.5 to 31.3. Almost three-fourths of the total students were categorized as "normal", while BMI of 26% was above normal. Participants with PCOS had higher BMI than those without it; however, no significant difference was found.

As evident from the table 3, the mean values of WHR, PBF, FFM, and VFA were found to be higher in participants with PCOS in contrast to those without PCOS. However, statistically significant difference could not be established at p<0.05.

BMD of the participants, on an average, was 2.3 ± 0.28 g/cm2 and it ranged from 1.8 to 3.1 ± 0.28 g/cm2. There was no significant difference between the BMD of those with PCOS and those without it at p<0.05.

**Table 2:** Medical history of participants related to PCOS (n=50)


Variable	Participants %

**Status of menstrual cycle**
Normal	80(40)
Oligomenorrhea	16(8)
Polymenorrhea	4 (2)
**Relevant diseases**	
None	84(42)
Glucose intolerance	2 (1)
Other	14(7)
**Use of hormonal pills**	
No	88(44)
Yes	12(6)
**Previous diagnosis of PCOS**	
No	76(38)
Yes	8 (4)
Doesn’t know	16(8)
**Family history of PCOS**	
No	76(38)
Yes	22(11)
Doesn’t know	2 (1)
**Perception of own body weight**	
Normal	64(32)
Underweight	14(7)
Overweight	18(9)
Obese	4 (2)
**Weight loss attempt**	
No	52(26)
Yes	48(24)


PCOS; Polycystic ovary syndrome.

**Table 3:** Means and standard deviations of body composition variables in participants with PCOS and those without PCOS


Variables	With PCOS	Without PCOS	t	P value

**Weight (kg)**	66.75± 14.4	58.85± 9.7	2.08	0.043*
**Body mass index (kg/m^2^)**	24.15± 3.9	22.65± 3.43	1.21	0.230
**Waist hip ratio**	0. 87 ± 0.06	0.845± 0.04	1.82	0.076
**Percent body fat (%)**	36.85± 8.7	33.75± 6.8	1.20	0.235
**Fat free mass (kg)**	48.15± 12.4	43.15± 10.6	1.28	0.207
**Visceral fat area (cm^2^)**	77.65± 26.3	64.95± 28.0	1.29	0.202
**Bone mineral density (g/cm^2^)**	2.315± 0.21	2.335± 0.30	0.173	0.863


*; Significant at p<0.05 and PCOS; Polycystic ovary syndrome.

## Discussion

Polycystic ovary syndrome is the most common endocrine disturbance that affects women. The aim of this study was to assess the occurrence of PCOS and its association with body composition among students in University of Sharjah. Our study reported that oligomenorrhea occurred in 16% of female students. Avvad et al. (16) reported that the menstrual irregularity in the early postmenarchal years may be an early sign of PCOS. Kitzinger and Wilmot (17) supported the presence of either irregular, absent or disrupted periods in women. van Hooff et al. (18) suggested that about 50% of the oligomenorrhic adolescents will develop PCOS as adults. A positive family history has been indicated in PCOS (19). Comparatively higher figures have been reported in earlier studies, 35% in mothers and 40% in sisters (20). Body dissatisfaction is observed to a greater extent in females suffering from PCOS. Himelein and Thatcher (21) as well as Trent et al. (22) confirmed that the common symptoms in PCOS (menstrual irregularities, obesity, male-pattern facial and body hair, acne, and other skin problems) contributed to poor body image and self-esteem and correlated with low quality-of-life scores. Moran et al. (23) concluded that there are potential barriers to successful weight management in young women who do not suffer from PCOS and additional barriers in women having PCOS.

The prevalence of PCOS (20%), based on our ultrasound findings, is consistent with those of other studies reporting prevalence of PCOS (8-33%) in women of reproductive age (24, 25). One-fourths of the total subjects in the current study were found to have BMI above normal; however, there was no significant difference between the BMI of subjects with or without PCOS. Yucel et al. (26) also revealed similar findings. Eleftheriadou et al. (27) reported a slightly higher percentage of overweight adolescents with PCOS than those without it. The histogram of BMI distribution in participants was found to be skewed towards the left, though it was not statistically significant. Similar to the current findings, no significant differences were reported between females with PCOS and controls in terms of WHR (28), PBF (29) as well as BMD (30). Barber et al. stated that it was global obesity (weight in the current study) rather than the abnormal regional fat distribution (VFA and WHR values in the current study) that characterized the PCOS women (30). However, WHR value of PCOS women was reported significantly higher than that of control subjects (29).

## Conclusion

The prevalence of PCOS in the present study is consistent with the global occurrence. Comparatively, the body composition of PCOS females is different from the normal females in terms of favoring more body weight, body fat, WHR and BMI. On the other hand, BMD is lesser in PCOS females than their normal counterparts. However, the further studies are needed in the Middle East region on larger sample sizes and broader aspects of health including the lifestyle and dietary components to understand the differences in weight in females suffering from PCOS.
